# Meta-analysis of structural and functional brain alterations in internet gaming disorder

**DOI:** 10.3389/fpsyt.2022.1029344

**Published:** 2022-10-25

**Authors:** Xiaoyu Niu, Xinyu Gao, Mengzhe Zhang, Zhengui Yang, Miaomiao Yu, Weijian Wang, Yarui Wei, Jingliang Cheng, Shaoqiang Han, Yong Zhang

**Affiliations:** ^1^Department of Magnetic Resonance Imaging, The First Affiliated Hospital of Zhengzhou University, Zhengzhou, China; ^2^Henan Key Laboratory of Magnetic Resonance and Brain Function, Zhengzhou, China; ^3^Henan Engineering Technology Research Center for Detection and Application of Brain Function, Zhengzhou, China; ^4^Key Laboratory for Functional Magnetic Resonance Imaging and Molecular Imaging of Henan Province, Zhengzhou, China; ^5^Henan Engineering Research Center of Medical Imaging Intelligent Diagnosis and Treatment, Zhengzhou, China; ^6^Zhengzhou Key Laboratory of Brain Function and Cognitive Magnetic Resonance Imaging, Zhengzhou, China; ^7^Henan Key Laboratory of Imaging Intelligence Research, Zhengzhou, China; ^8^Henan Engineering Research Center of Brain Function Development and Application, Zhengzhou, China

**Keywords:** voxel-based morphometry, functional magnetic resonance imaging, meta-analysis, internet gaming disorder, gray matter volume

## Abstract

**Background:**

Many neuroimaging studies have reported abnormalities in brain structure and function in internet gaming disorder (IGD). However, the findings were divergent. We aimed to provide evidence-based evidence of structural and functional changes in IGD by conducting a meta-analysis integrating these studies quantitatively.

**Method:**

A systematic search was conducted in PubMed, ScienceDirect, Web of Science, and Scopus from January 1, 2010 to October 31, 2021, to identify eligible voxel-based morphometry (VBM) and functional magnetic resonance imaging (fMRI) studies. Brain alternations between IGD subjects and healthy controls (HCs) were compared using the anisotropic seed-based d mapping (AES-SDM) meta-analytic method. Meta-regression analysis was used to investigate the relationship between gray matter volume (GMV) alterations and addiction-related clinical features.

**Results:**

The meta-analysis contained 15 VBM studies (422 IGD patients and 354 HCs) and 30 task-state fMRI studies (617 IGD patients and 550 HCs). Compared with HCs, IGD subjects showed: (1) reduced GMV in the bilateral anterior/median cingulate cortex, superior/inferior frontal gyrus and supplementary motor area; (2) hyperactivation in the bilateral inferior frontal gyrus, precentral gyrus, left precuneus, right inferior temporal gyrus and right fusiform; (3) hypoactivation in the bilateral lingual and the left middle frontal gyrus; and (4) both decreased GMV and increased activation in the left anterior cingulate. Furthermore, Meta-regression revealed that GMV reduction in left anterior cingulate were positively correlated with BIS-11 score [*r* = 0.725, *p* = 0.012(uncorrected)] and IAT score [*r* = 0.761, *p* = 0.017(uncorrected)].

**Conclusion:**

This meta-analysis showed structural and functional impairments in brain regions related to executive control, cognitive function and reward-based decision making in IGD. Furthermore, multi-domain assessments captured different aspects of neuronal changes in IGD, which may help develop effective interventions as potential therapeutic targets.

## Introduction

Internet addiction has aroused widespread concern around the world and led to many related mental diseases (1). Internet gaming disorder (IGD) accounted for 57.5% of all types of Internet addiction among college students (2) defined as the inability of an individual to control his/her use of online game behaviors, which has many similarities with pathological gambling (3–5). Many players exhibit symptoms such as decreased executive function (6), excessive impulsivity (7, 8), impaired risky decision-making ability (9) and craving (10, 11). Although unlike substance addiction, IGD does not consume addictive substances, it can lead to dependence and mental and physical health problems similar to other addictions due to excessive gaming (12, 13). Given its growing prevalence and negative effects, IGD has been listed in Section “Results” of the fifth edition of the Diagnostic and Statistical Manual for Mental Disorders (DSM-5) as a condition worthy of further research. Therefore, more evidence is needed to understand the neuropathic factors behind IGD, which will facilitate future research and shed light on the success of its treatment.

As a non-invasive imaging technique, magnetic resonance (MR) imaging has shown great potential value in elucidating the neuropathogenesis of psychiatric disorders. Functional magnetic resonance imaging (fMRI) has been widely used to reveal neural changes in addictive disorders (14–17). Previous studies in functional neuroimaging (12, 18–20) have revealed that during performing the impulse control-related tasks, IGD subjects had aberrant activations in the frontal, insular, temporal and parietal cortex compared with the HCs. In addition to functional activity changes, gray matter volume (GMV) as a structural marker of the brain may be relatively stable over time and can be used as a basis for functional neural activity (21). GMV has been widely used in many studies of mental disorders (22–24). VBM is a voxel-based gray matter volume measurement method that can detect subtle changes in GMV. Previous VBM studies have demonstrated that IGD patients have reduced GMV in brain regions associated with executive control such as the anterior cingulate cortex (ACC) and dorsolateral prefrontal cortex involved in processing goal-directed behaviors (5, 11, 25, 26). Therefore, a quantitative synthesis of VBM studies in IGD can provide additional information to complement the results of fMRI studies. The combination of different modalities can more comprehensively identify the common and specific neural changes in IGD. The severity of IGD was measured by Young’s Internet addiction test (IAT). IAT was a reliable and valid instrument for classifying Internet addiction disorder (27). The clinical relevance of impulsivity was frequently highlighted because it affected many mental and behavioral disorders. Cao and Lee have found that IGD subjects were more impulsive than healthy controls (8, 28), which may lead to serious impairments in psychological and social functions, such as suicide attempts and crime. The severity of Internet addiction disorder (IAD) was positively correlated with both behavioral impulsivity and self-reported impulsivity (28). Barratt Impulsiveness Scale-11 (BIS-11) score could well assess the core impulsivity characteristics of addiction (29).

Although many neuroimaging studies have shown changes in brain structure and function in patients with IGD, these findings are inconsistent or even contrary. Consequently, it is of great significance to perform a pooled meta-analysis to provide evidence-based evidence of structural and functional changes in IGD by pooling “observations” with controls for random effect. A preliminary meta-analysis of 10 VBM studies and 27 fMRI studies has found brain hyperactivation in the anterior and posterior cingulate cortices, caudate and posterior inferior frontal gyrus (IFG), hypoactivation in the anterior IFG, the posterior insula, and reduced gray-matter volume in the anterior cingulate, orbitofrontal and dorsolateral prefrontal (30). However, in recent years, there have been substantial novel and high-quality studies on this subject. Compared with previous meta-analysis, this study not only included more new studies to explore the neural changes of IGD, but also focused on exploring the relationship between brain GMV and impulsivity in IGD patients. It is high time that we performed an updated meta-analysis to confirm, supplement, and/or modify the results of previous meta-analysis.

The purpose of our study was to conduct two meta-analyses separately including numerous proven VBM and fMRI studies and a conjunction analysis between two meta-analyses to explore: (a) brain GMV and functional abnormalities; (b) the association between some common addiction-related clinical features and GMV alterations. Based on previous empirical studies, we hypothesized that IGD subjects compared to HCs would show GMV and functional alterations in brain regions involved in reward-based decision-making such as the inferior prefrontal cortex (31), cognitive control such as the cingulate gyrus and the precuneus (18), and visual cognitive functions such as the fusiform (8). As a reliable structural marker, GMV can easily identify different psychiatric disorders without potentially confounding considerations of tasks in fMRI (32). Therefore, we also hypothesized that the GMV alterations in IGD subjects would be closely associated with clinical variables such as the IAT score and BIS-11 score.

## Methods

### Inclusion of studies

Extensive searches were carried out in PubMed, ScienceDirect, Web of Science, and Scopus from January 1, 2010, to October 31, 2021, combined with the following keywords: (“voxel-based morphometry” or “VBM” or “gray matter” or “voxel-wise” or “functional magnetic resonance imaging” or “fMRI”) and (“online-game” or “Internet gaming disorder” or “IGD”). Studies were included if (1) they used specific tasks during the MRI scan or they used VBM to analyze gray matter; (2) they provided whole-brain pairwise voxel-based comparisons of patient groups (IGD) relative to controls; (3) the studies reported Montreal Neurological Institute (MNI) or Talairach coordinates of the whole brain; (4) there were no neurological or psychiatric comorbidities such as depression, anxiety, autism, learning disorder and epilepsy; (5) studies were peer-reviewed and published in English. Studies were excluded if (1) they only reported region of interest findings; (2) peak coordinates were still not available even if we contacted the authors by email; (3) studies used tensor-based morphometry; (4) they were unpublished studies; (5) they didn’t use the same threshold throughout the whole brain within each included study. Three authors independently searched, selected, and cross-checked the literature. Any divergence was settled through a joint revaluation of the original studies. And then we implemented the following steps.

### Statistical analysis

Structural and functional brain differences between individuals with IGD and healthy controls were analyzed by using the anisotropic seed-based d mapping (AES-SDM) meta-analytic software, version 5.15^1^. The AES-SDM method has been well validated in recent meta-analysis of psychiatric diseases (33, 34). AES-SDM is a statistical technique that can use previously reported peak coordinates and effect sizes (*t*-scores) to calculate signed (positive/negative) effect sizes and variance maps of brain regional differences between patient and control groups. For each meta-analysis, maps are combined across studies based on the random-effect model, considering sample size, intra-study variability and inter-study heterogeneity (35). The processing process of AES-SDM data is summarized here^2^. Here we briefly outline the steps: (1) we extracted peak coordinates and effect sizes (*t*-values) from each included study. Sometimes some studies included z-scores without *t*-values, then the z-scores could be converted to t statistics using an online converter^3^; (2) we converted peak coordinates into standardized MNI space; (3) we set the full width at half maximum (FWHM) to 20 mm as this will maintain a balance between sensitivity. Voxel *p* < 0.005 was used as a significant threshold. Peak height threshold > 1 and cluster extent threshold > 10 voxels were supplemented to optimally balance sensitivity and specificity (36).

First, two meta-analyses including all fMRI and VBM studies were conducted separately to identify neural changes in IGD. Second, a multimodal analysis was further performed on patients and controls to examine overlapping regions of functional and structural abnormalities. Next, a jack-knife sensitivity analysis was also conducted to test the robustness of the results by repeating the same analysis excluding one study each time and to assess the reproducibility of the results for each meta-analysis (36). If one brain region survives in most of the repeats, we consider the finding to be highly replicable. Then meta-regression analysis was conducted to explore the association between GMV alterations and clinical features including the BIS-11 score and IAT score. At last, funnel plots constructed by AES-SDM (Supplementary Figure 1) and Egger’s test were used to examine possible publication bias (37).

## Results

### Included studies and sample characteristics

**FIGURE 1:**
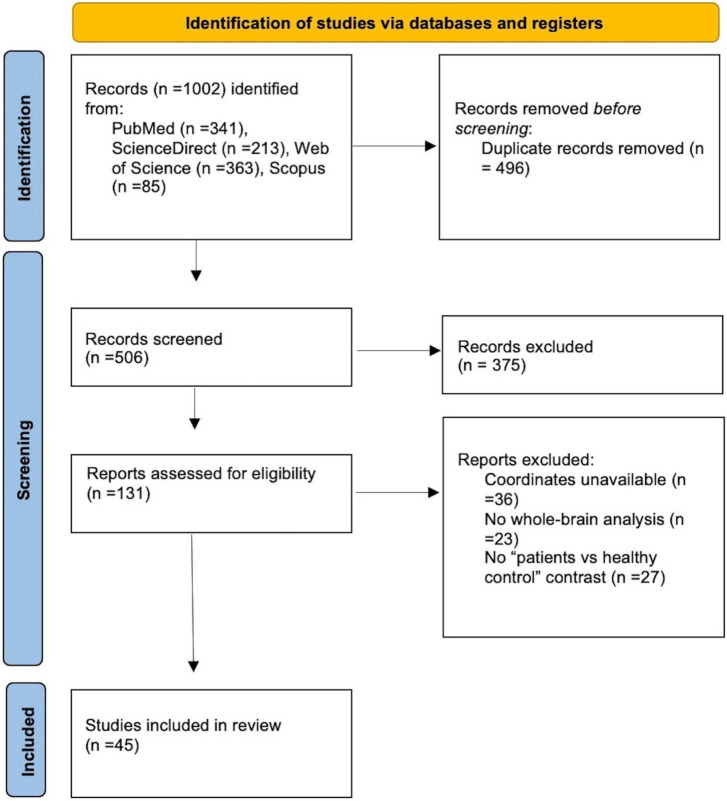
Prisma flow diagram for including eligible studies in the meta-analysis.

Searching in various databases, a total of 1002 records were identified. After removing duplicates, 506 records were screened and 131 full-text articles were assessed for eligibility. Our final dataset consisted of 15 VBM studies including 422 IGDs and 354 HCs and 30 fMRI studies including 617 IGDs and 550 HCs (Figure 1 and Table 1).

**TABLE 1:** Demographic and clinical characteristics of voxel-based morphometry and functional magnetic resonance imaging studies in internet gaming disorder.

References	Age group	Patients		Controls		Tasks	Multiple comparisons	BIS-II	IAT
			
		Number (% male)	Mean age, y	Number (% male)	Mean age, y				
**(1) VBM studies**									
Du et al. (38)	Adolescents	25 (100)	17.28	27 (100)	17.48	NA	Uncorr.	68.56 (55.33)	69.96 (32.15)
Han et al. (4)	Adults	20 (100)	20.90	18 (100)	20.90	NA	FDR	61.5 (50.1)	NA
He et al. (39)	Adults	26 (77) 20	20.69	26 (77)	20.46	NA	few	NA	NA
Jin et al. (40)	Adults	25 (64) 16	19.12	21 (67)	18.76	NA	few	63.28 (29.19)	63.28 (26.19)
Ko et al. (41)	Adults	30 (100)	23.57	30 (100)	24.23	NA	FDR	78.50 (62.87)	NA
Lee et al. (42)	Adults	31 (100)	24.00	30 (100)	23.00	NA	few	54.4 (45.2)	64.4 (31.5)
Lee et al. (43)	Adults	20 (100)	23.90	20 (100)	22.70	NA	Uncorr.	51.6 (47.4)	58.6 (31.3)
Lin et al. (44)	Adults	35 (100)	22.28	36 (100)	22.20	NA	FDR	NA	72 (29)
Mohammadi et al. (45)	Adults	29 (100)	23.60	29 (100)	22.70	NA	few	NA	NA
Seok et al. (46)	Adults	20 (100)	21.70	20 (100)	22.40	NA	FDR	56.00 (47.50)	71.85 (29.80)
Sun et al. (47)	Adults	18 (83) 15	20.50	21 (86)	21.95	NA	AlphaSim	63.94 (50.81)	NA
Weng et al. (48)	Adolescents	17 (24) 4	16.25	17 (12)	15.54	NA	few	68.85 (65.94)	NA
Yoon et al. (49)	Adults	19 (100)	22.90	25 (100)	25.40	NA	Cluster-size inferences	70.10 (54.3)	76.3 (26.0)
Zhou et al. (17)	Adolescents	18 (89) 16	17.23	15 (87)	17.81	NA	FDR	NA	NA
Lee et al. (50)	Adults	18 (100)	25.40	18 (100)	25.80	NA	Uncorr.	58.8 (48.8)	72.4 (32.1)
**(2) fMRI studies**									
Chiao et al. (51)	Adults	15 (100)	24.67	15 (100)	24.47	Go/No-Go task	FWE	74.33 (62)	NA
Chun et al. (52)	Adolescents	16 (100)	13.60	19 (100)	13.37	Cue-reactivity task	FDR	NA	NA
Dieter et al. (53)	Adults	15 (87)	28.70	17 (76)	24.94	Self-perception task	FWE	NA	NA
Ding et al. (54)	Adolescents	17 (82)	16.40	17 (82)	16.29	Go/No-Go task	AlphaSim	62.71	NA
Dong et al. (55)	Adults	18 (100)	21.00	21 (100)	22.00	Stroop task/guessing task	Uncorr.	NA	79.5
Dong et al. (56)	Adults	16 (100)	21.40	15 (100)	22.10	Wins-and-losses task	FWE	NA	NA
Dong et al. (57)	Adults	14 (100)	23.40	13 (100)	24.10	Guessing task	FDR	NA	NA
Dong et al. (58)	Adults	20 (100)	21.30	16 (100)	21.90	Risk-taking/risky decision-making task	AlphaSim	NA	NA
Dong et al. (19)	Adults	15 (100)	21.60	15 (100)	22.40	Color–word Stroop task	Uncorr.	NA	NA
Han et al. (59)	Adolescents	16 (100)	14.20	15 (100)	14.00	Cue-reactivity task	FDR	NA	NA
Ko et al. (5)	Adults	15 (100)	24.70	15 (100)	24.47	Cue-reactivity task	Uncorr.	NA	NA
Ko et al. (10)	Adults	26 (100)	24.60	23 (100)	24.35	Go/No-go task	FDR	79.08	NA
Lee et al. (60)	Adults	24 (100)	24.80	24 (100)	24.30	Risky decision-making task	Uncorr.	25.8 (22.7)	50.9 (27.5)
Lee et al. (61)	Adolescents	18 (18)	13.60	18 (100)	13.40	Stroop Match-to-Sample task	Uncorr.	65.1 (18.4)	NA
Leménager et al. (62)	Adults	16 (88)	28.30	17 (76)	24.94	Self-perception task	FWE	NA	NA
Lin et al. (20)	Adults	19 (100)	22.20	21 (100)	22.80	Probability–discounting task	Uncorr.	NA	NA
Liu et al. (63)	Adults	39 (100)	22.60	23 (100)	23.09	Cue-reactivity task	FWE	NA	NA
Liu et al. (64)	Adults	11 (100)	23.50	11 (100)	22.45	Go/no-go task	Uncorr.	NA	NA
Liu et al. (11)	Adults	41 (100)	21.90	27 (100)	22.74	Cups task	FWE	NA	NA
Lorenz et al. (65)	Adults	8 (100)	25.00	9 (100)	24.80	Dot probe paradigm/cue reactivity task	AlphaSim	74.9 (70.6)	NA
Ma et al. (66)	Adults	29 (100)	22.60	23 (100)	23.09	Cue-reactivity task	FWE	NA	NA
Qi et al. (67)	Adolescents	23 (100)	17.30	24 (100)	17.42	Balloon analog risk task	AlphaSim	68.17 (54.13)	70.35 (33.42)
Qi et al. (68)	Adolescents	24 (100)	17.20	24 (100)	17.42	Balloon analog risk task	AlphaSim	68.79 (54.13)	70.71 (33.42)
Shin et al. (69)	Adults	20 (x)	22.10	21 (x)	22.14	Go/no-go task	FWE	51.25 (38.57)	76 (26.71)
Sun et al. (70)	Adults	10 (100)	20.40	10 (100)	20.30	Cue-reactivity task	FDR	NA	NA
Wang et al. (71)	Adults	20 (100)	21.00	20 (100)	21.95	Delay discounting task/probabilistic discounting task	FWE	NA	65.55 (31)
Zhang et al. (72)	Adults	19 (100)	22.20	21 (21)	22.80	Addiction Stroop task	FWE	NA	NA
Zhang et al. (73)	Adults	40 (100)	22.00	19 (100)	22.89	Cue-reactivity task	GRFT	NA	NA
Turel et al. (74)	Adults	26 (77) 20	20.46	26 (77) 20	20.69	Cue-reactivity task	FWE	NA	NA
Wang et al. (75)	Adults	27 (100)	22.52	26 (100)	23.23	Roulette task	FDR	NA	70.15 (22.31)

WHO, Children, 1−9 years; Adolescents, 10−19 years; Adults, > 19 years; BIS-11, Barratt Impulsiveness Scale-11; IAT, internet addiction test.

### Meta-analysis result

#### Voxel-based morphometry meta-analysis

**FIGURE 2:**
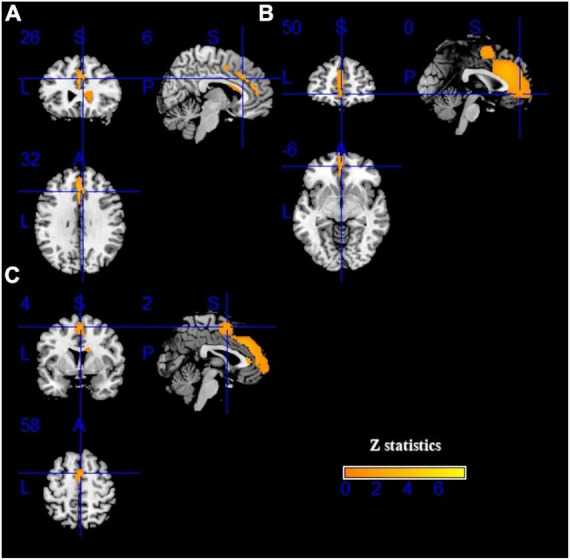
Results of voxel-based morphometry (VBM) for internet gaming disorder (IGD). Gray matter volume (GMV) reductions were displayed in yellow shown in the sagittal, axial, and coronal planes separately. **(A–C)** show three different brain regions.

As demonstrated in Figure 2 and Table 2, IGD subjects showed significantly smaller GMV in the bilateral anterior cingulate cortex (ACC), median cingulate cortex (MCC), superior frontal gyrus, medial orbital (SFG), inferior frontal gyrus (IFG) and supplementary motor area (SMA) compared with healthy controls. Compared with the control group, there was no significant increase of GMV in the IGD group.

**TABLE 2:** Meta-analysis results across all studies.

Region		MNI coordinate	SDM Z score	*P*-value	No. of voxels	Breakdown (no. of voxels)	Jack-knife sensitivity	Brodmann areas
**(1) VBM RESULTS**								
Clusters of decreased GMV	R median cingulate/paracingulate gyri	6, 26, 32	−3.479	0.000077605	1387		15 out of 15	32,24,10,11
						L ACC (399)		
						R ACC (67)		
						L MCC (100)		
						R MCC (35)		
	L superior/inferior frontal gyrus, medial orbital	0, 50, −6	−1.785	0.000077605	647		12 out of 15	32,10,11,9,8,24
						R S/IFG (59)		
	L/R supplementary motor area	2, 4, 58	−2.165	0.000007033	331		10 out of 15	6,32,24
						L SMA (199)		
						R SMA (125)		
**(2) fMRI RESULTS**								
IGDs > HCs	Inferior frontal gyrus, opercular part	42, 8, 24	2.727	∼0	1404		26 out of 30	48,44,45,6
						R IFG, opercular part (458)		
						R IFG, triangular part (277)		
						R precentral gyrus (213)		
						R MFG (31)		
	L inferior frontal gyrus, triangular part	−46, 14, 30	2.848	∼0	1029		26 out of 30	44,48,6
						L IFG, opercular part (326)		
						L precentral gyrus (247)		
						L IFG, triangular part (99)		
						L insula (40)		
	L precuneus	−4, −60, 46	2.174	0.000701845	274		28 out of 30	7,5
						L precuneus (229)		
						R precuneus (44)		
	L/R ACC	4, 10, 26	1.952	0.002771378	66		25 out of 30	24
						L ACC (49)		
						L median network, cingulum (22)		
						R ACC (17)		
	R supramarginal	58, −24, 32	1.899	0.003777742	29		26 out of 30	48,2
	R inferior temporal gyrus	54, −44, −16	1.919	0.003375173	13		25 out of 30	20
	R fusiform	32, −76, −14	1.985	0.002373993	11		26 out of 30	19
IGDs < HCs	L calcarine	0, −82, −8	−1.216	0.001140535	478		27 out 30	17
						L calcarine (64)		
						R calcarine (19)		
	L/R lingual	−4, −80, −2	−1.164	0.001522422	215		30 out of 30	17,18
	L middle frontal gyrus	−24, −4, 48	−1.157	0.001599848	19		26 out of 30	6

L, Left; R, right; MNI, Montreal Neurological Institute; ACC, anterior cingulate cortex; MCC, median cingulate cortex; IFG, inferior frontal gyrus; SFG, superior frontal gyrus; SMA, supplementary motor area; MFG, middle frontal gyrus; FWE-corrected *p* < 0.05.

#### Functional magnetic resonance imaging meta-analysis

**FIGURE 3:**
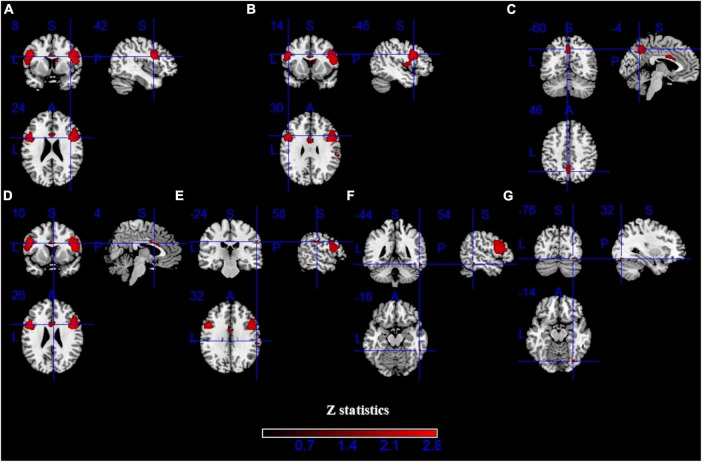
Results of functional magnetic resonance imaging (fMRI) for IGD. Clusters with hyperactivation were shown in red. **(A–G)** show seven different brain regions.

**FIGURE 4:**
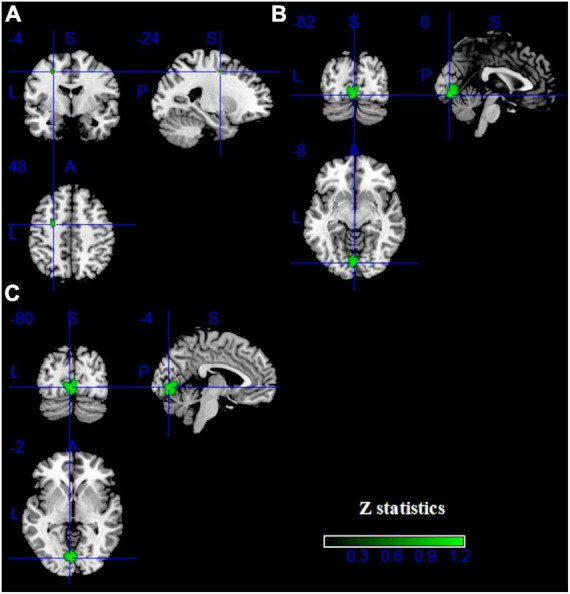
Results of fMRI for IGD. Clusters with hypoactivation were shown in green. **(A–C)** show three different brain regions.

Pooling across all fMRI studies, IGDs showed significantly higher activation in the bilateral inferior frontal gyrus (IFG), precentral gyrus, left precuneus, right inferior temporal gyrus (ITG), right supramarginal and right fusiform compared with HCs (Figure 3 and Table 2). Besides, lower activation was also detected in the bilateral lingual, calcarine and left middle frontal gyrus (MFG) (Figure 4 and Table 2).

#### Multimodal voxel-based morphometry and functional magnetic resonance imaging analysis

**FIGURE 5:**
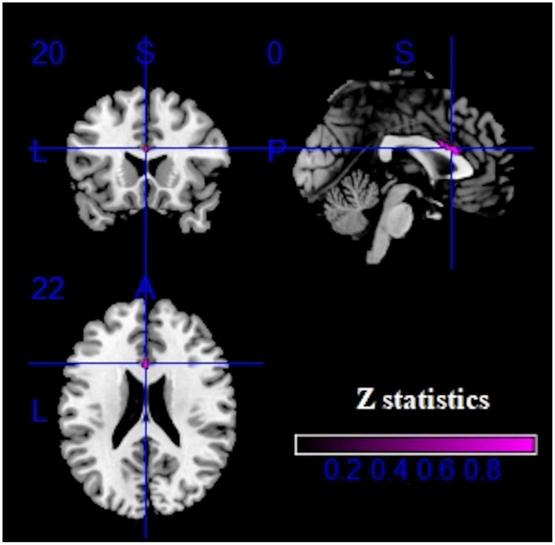
Multimodal analysis results in the IGD. Purple clusters represent reduced GMV and increased brain activation.

Compared with healthy controls, IGD subjects showed both decreased GMV and increased activation in the left ACC (MNI coordinates, 0, 20, 22; 15 voxels) (Figure 5).

### Reliability analyses

For the VBM meta-analysis, a systematic whole-brain jackknife sensitivity analysis revealed a highly robust GMV decrease in bilateral anterior cingulate cortex (ACC) and median cingulate cortex (MCC) because these results were replicable in all 15 datasets. Alterations in the supplementary motor area (SMA) and inferior frontal gyrus (IFG) remained highly replicable, as they could be identified in at least 12 and 10 of the 15 combinations respectively. For the fMRI meta-analysis, hypoactivation of bilateral lingual was highly replicable, as these findings were preserved throughout all 30 combinations of datasets. Besides, the results in the left calcarine and left middle frontal gyrus were also significant because they could be identified in 27 and 26 datasets respectively. Hyperactivation of bilateral inferior frontal gyrus, right supramarginal, right fusiform, left precuneus and right inferior temporal gyrus (ITG) remained robust as at least 25 of the 30 combinations were identifiable (Table 2).

### Meta-regression

**FIGURE 6:**
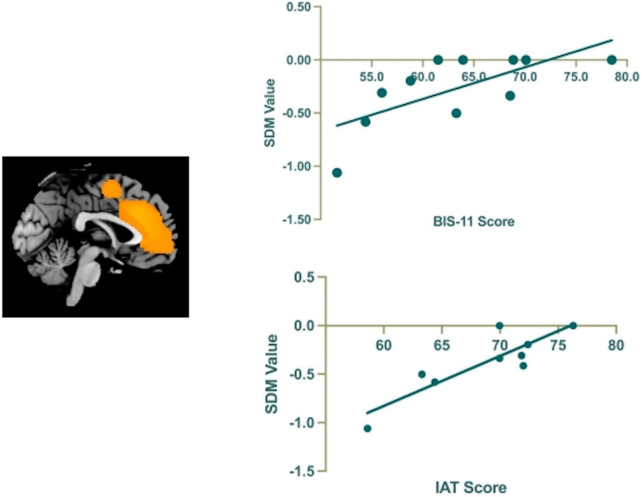
The results of the meta-regression analysis show that the BIS-11 and IAT scores are positively correlated to regional GMV reduction in the left anterior cingulate cortex (ACC).

In our study, the variables explored by meta-regression analysis included the BIS-11 score and IAT score. The results showed that GMV reduction in left ACC (MNI coordinate, 0, 28, 22; SDM-Z, −3.067; *p* = 0.00001; 477 voxels) was positively correlated with BIS-11 score [*r* = 0.725, *p* = 0.012(uncorrected)] and IAT score [*r* = 0.761, *p* = 0.017(uncorrected)] (Figure 6).

## Discussion

Our study integrated the results of 15 VBM studies and 30 fMRI studies to explore changes in brain GMV and functional neural activation in IGD patients compared to healthy controls, using an anisotropic seed d-Mapping (AES-SDM) method. The result demonstrated that GMV reduction was found in the anterior cingulate cortex (ACC), median cingulate cortex (MCC), and supplementary motor area (SMA). Functionally, IGDs as compared with HCs showed significantly higher activation in the left precuneus, right inferior temporal gyrus (ITG), fusiform and inferior frontal gyrus (IFG). Besides, lower activation in the left middle frontal gyrus (MFG) and the lingual was also detected, stable and replicable under jack-knife sensitivity analysis. In addition, both decreased GMV and increased activation was found in the left ACC among IGD patients. Moreover, meta-regression analysis revealed that higher BIS-11 score and IAT score were correlated with decreased GMV in the left ACC. These findings could become a preliminary implication of neural structural and functional biomarkers in IGD and may help develop effective interventions in these brain regions as potential therapeutic neuro-target for IGD.

Consistent with previous findings in IGD (26, 76), robust GMV decrease in brain regions involved in executive control, namely, the ACC and the SMA (77). The ACC plays important roles in the neural activity of several large-scale networks (78) and is critically involved in multiple processes including cognitive control (79), emotional regulation (80), reward-based decision-making (81), conflict monitoring and error processing (82).

Unsurprisingly, the results of the VBM and fMRI meta-analyses converged on the ACC, showing both functional hyperactivation and gray-matter reduction in IGDs as compared to HCs, which is consistent with previous studies suggesting that structural or functional alterations in the region lead to impaired executive control (10, 83). A study by Wang et al. (76) showed that the GMV of the ACC was negatively correlated with conflict monitoring in the Stroop task. As a functional imaging study demonstrated, IGD individuals exhibited altered brain activity in the ACC during error processing (56). Although numerous executive control problems have been implicated in IGD as mentioned above, IGD is most consistently associated with high impulsivity (28). Conflict monitoring capacity is closely associated with impulse control (84). Our results showed that higher BIS-11 scores are significantly associated with decreased GMV in the left ACC, suggesting that high impulsivity in IGD patients may be due to structural abnormalities in the executive control regions of the brain (38, 76). Although the direction of the alteration of the ACC is inconsistent across modalities (fMRI and VBM), there is evidence that an increase or decrease in GMV may not simply correspond to functional neural activation or inactivation (85). Previous studies have been shown that the ACC always activates in the Stroop task involved in conflict monitoring and cognitive control (86–88). In conclusion, fMRI and VBM may reflect the distinctive aspects of neural alterations, it is plausible to postulate that the alterations of the ACC in IGD subjects may play a vital role in a dysfunctional interaction between executive control and reward-based decision making.

We find decreased GMV of SMA in IGD people through VBM analysis. The SMA is capable of controlling internally generated movements and action sequences (89). Spending more time playing computer games and on repetitive motor actions, such as clicking the mouse or hitting the keyboard, may cause structural changes in the SMA. Just like the cingulate gyrus, the SMA also contributes significantly to cognitive control (77) such as integrating sensory information and monitoring conflict (90). A study has shown that disruptions of the SMA can provoke impairments in response inhibition (91). Besides, during a Go/No-Go Task, IGD subjects showed decreased activation of the SMA for response inhibition compared with healthy controls in a functional imaging study (51). We speculate that IGD subjects’ inability to control their urge to play games is associated with a reduction in SMA gray matter involved in response inhibition, consistent with previous evidence that appropriate response inhibition may be a key aspect of impulse control (92).

The findings of lower activation of the MFG in IGD subjects cannot be ignored. It is commonly believed that the MFG contributes to inhibitory control (57, 83, 93). A study (58) has found that the MFG activation was negatively correlated with the Stroop effect in the IGD groups, which is consistent with previous studies on IGD subjects in go/no-go task (94) and switching (95) task. Hypoactivation in the MFG in IGD subjects probably suggests that they may engage less endeavor in controlling their impulses to play games.

The precuneus, also known as the posterior region of the medial parietal cortex, plays an important role in fundamental cognitive functions such as episodic memory retrieval, visual-spatial information, spatially guided behavior, and attentional processing (96). Numerous studies have confirmed that parietal areas are activated during attention-shifting and are activated by visual addiction-related cues when attention is reflexively drawn to salient features of the stimulus (97). Higher brain activation in precuneus in IGD subjects may indicate that they experience more cognitive conflict and require more top-down attention during the addictive Stroop task (98, 99). That means, gaming-related words have attracted IGD subjects’ attention during the addiction Stroop task and the precuneus was activated to promote cue-induced cravings for online gaming.

The fusiform, located in the middle of the ventral temporal lobe, is considered to be one of the most important brain regions in the visual ventral stream and plays an important role in a range of visual cognitive functions, such as the recognition of the face, body and various object features (100–104), color information processing and emotion perception in facial stimulation (105). Furthermore, the fusiform topographically connects the striate cortex to the inferior temporal lobe, which is associated with auditory processing, comprehension and verbal memory (47). We observed significant activation of the fusiform and inferior temporal gyrus, which may indicate that IGD subjects were more focused on the visual and auditory stimuli of games and were reminded of past online gaming experiences, triggering cravings.

What’s more, we also found increased activation in the inferior frontal gyrus (IFG) involved in risk-evaluation (106, 107) and audio-visual information accumulation in decision-making tasks (108), as well as in the regulation between reward and risk levels (20, 106). A study by Dong et al. (56) demonstrated that IGD subjects needed longer time than healthy controls to make decisions and showed greater activation of the inferior frontal gyrus both in WIN and LOSS trials. Another research found that the activation of the IFG had a positive correlation with the risk aversion (106). We speculate the reason for hyperactivation in IFG is that IGD subjects need more endeavors to complete the decision-making task and may affect executive functions needed to perform other tasks such as conflict inhibition. It could also explain that the occurrence of negative outcomes can lead to further negative reinforcement and the continuation of addictive behaviors to avoid negative effects (109).

## Limitation

This meta-analysis sheds new light on brain structure and functional changes of IGD, which may have implications for both clinical interventions and future research. However, there are still some limitations that need to be considered. First, it is difficult to rule out the inter-study heterogeneity of methodologies (including MRI machine, slice thickness, pretreatment protocol, and statistical threshold), which may influence our results. Future research should focus on these issues. Second, the meta-analysis was based primarily on peak coordinates rather than raw statistical brain maps, it may not be sufficient enough to detect some results with small or medium effect sizes (30, 110). Third, male participants were dominant in all samples, and we did not analyze neurological differences in IGD patients of different genders. Fourth, due to the integration of cross-sectional studies, the causal relationship between the structural and functional brain changes and the development of addiction still requires careful consideration. As a consequence, longitudinal studies should be focused on in future research.

## Conclusion

In summary, our pooled meta-analysis found distinctive brain structural and functional alterations in IGD across different modalities. Evidence from brain functional abnormalities and gray-matter volume alteration converged to show that IGD was associated with brain regions involved in executive control, cognitive function, and reward-based decision-making such as the ACC, the SMA, the precuneus, fusiform and the IFG. Meta-regression analysis further explored the association between brain structural alterations in IGD and clinical information. Our findings stress the neurofunctional and neurostructural biomarkers for IGD, which may help to develop effective interventions for these brain regions as potential behavioral, pharmacological or neurotherapeutic targets. Besides, longitudinal studies should be performed to complement and validate our findings in the future.

## Data availability statement

The original contributions presented in this study are included in the article/Supplementary material, further inquiries can be directed to the corresponding author/s.

## Author contributions

XN, MZ, XG, ZY, MY, and YZ conceived and designed the study. XN analyzed the data, performed the statistical study, and drafted the manuscript. MZ, YZ, JC, SH, and XG revised the manuscript. All authors contributed to the article and approved the submitted version.

## Footnotes

1
https://www.sdmproject.com/

2
http://www.sdmproject.com/software/tutorial.pdf

3
http://www.sdmproject.com/utilities/?show=Statistics

## Supplementary material

Supplementary Figure 1 Funnel plots for significant results.
