# Supplementary figures and images for: Meta-analysis of structural and functional brain alterations in internet gaming disorder
Source: Front Psychiatry. 2022 Oct 25;13:1029344. doi: 10.3389/fpsyt.2022.1029344 (PMC10074425; doi:10.3389/fpsyt.2022.1029344)

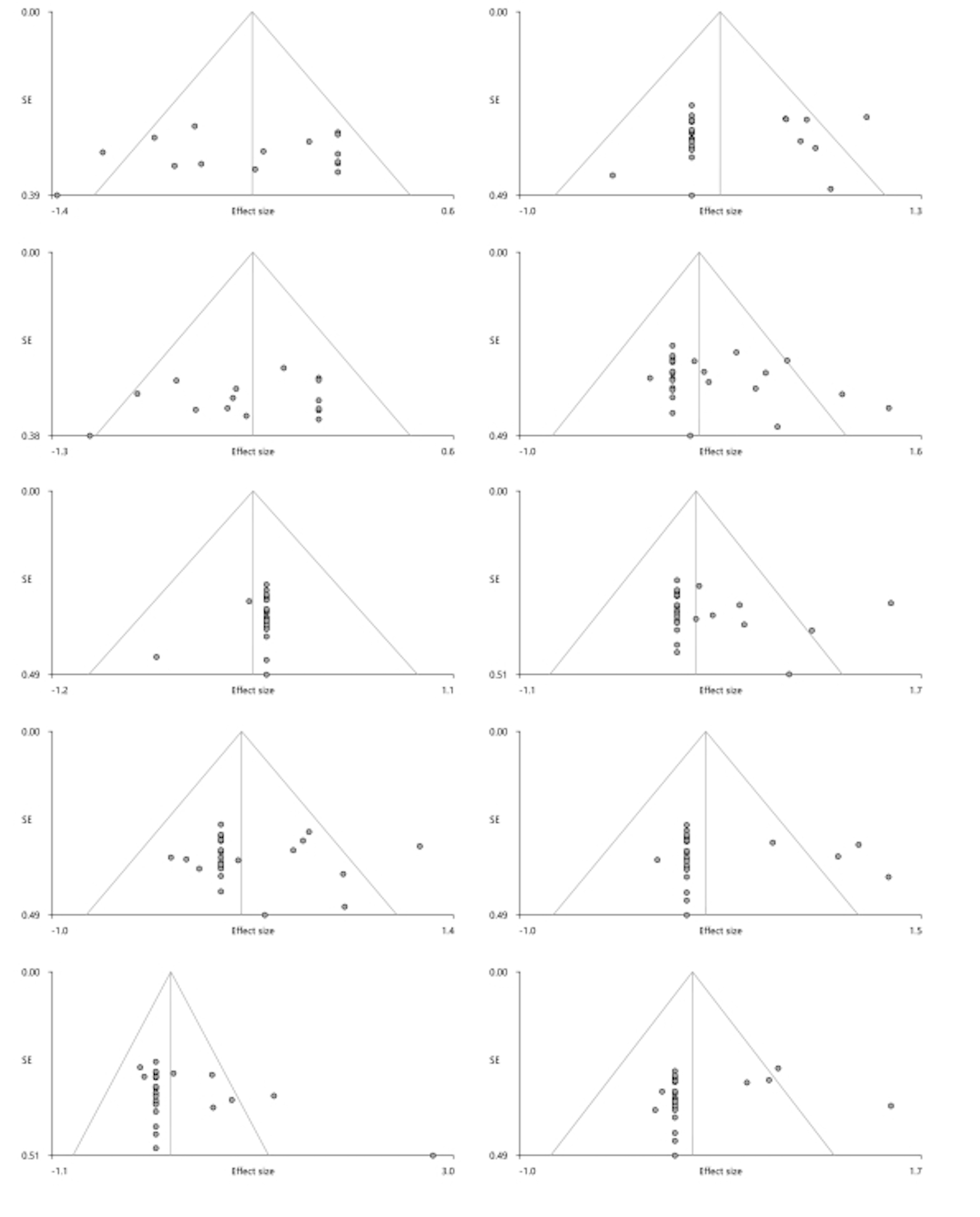

Supplement: Supplementary Figure 1 — Funnel plots for significant results. [file Image_1.JPEG]
